# Advanced Maxillary Squamous Cell Carcinoma With Extension to the Infratemporal Fossa Masquerading as Extensive Osteomyelitis: A Case Report

**DOI:** 10.7759/cureus.113503

**Published:** 2026-07-28

**Authors:** Snehasree Das, Ashok Kumar KR, Kotresh N S, Praveen KS, Kokila Ganganna

**Affiliations:** 1 Oral and Maxillofacial Surgery, Sri Siddhartha Dental College and Hospital, Tumkur, IND; 2 Radio Diagnosis, Sri Siddhartha Medical College, Tumkur, IND; 3 Oral and Maxillofacial Pathology, Sri Siddhartha Dental College and Hospital, Tumkur, IND

**Keywords:** chemotherapy, incisional biopsy, infratemporal fossa extension, maxillary osteomyelitis, maxillary sinus, maxillary squamous cell carcinoma, oral squamous cell carcinoma

## Abstract

Squamous cell carcinoma (SCC) of the maxilla is an uncommon malignancy that often presents at an advanced stage because of its nonspecific clinical features and proximity to adjacent anatomical structures. In rare instances, it may closely mimic chronic osteomyelitis, particularly when presenting as a non-healing extraction socket with exposed necrotic bone and extensive osteolytic destruction, leading to delayed diagnosis and treatment. We report a diagnostically challenging case of advanced maxillary SCC that initially masqueraded as extensive osteomyelitis. A 55-year-old female with a 20-year history of areca nut chewing presented with a three-month history of pain, swelling, and purulent discharge from the left posterior maxilla following extraction of a mobile maxillary molar four months earlier. Clinical examination revealed diffuse left midfacial swelling, mild restriction in mouth opening, and an ulcerative lesion involving the left maxillary tuberosity, posterior alveolar ridge, and hard palate, with exposed necrotic bone and palatal perforation. Contrast-enhanced computed tomography demonstrated extensive osteolytic destruction involving the hard palate, all walls of the left maxillary sinus, left pterygoid bone, left lateral wall of the nasal cavity, medial wall of the right maxillary sinus, and extension into the left infratemporal fossa, with imaging findings suggestive of extensive osteomyelitis. Incisional biopsy revealed well-differentiated SCC. Following multidisciplinary evaluation, the patient was treated with three cycles of induction chemotherapy using the docetaxel, cisplatin, and 5-fluorouracil (TPF) regimen, resulting in partial clinical improvement, and she continues under oncologic follow-up. Advanced maxillary SCC may closely mimic chronic osteomyelitis both clinically and radiologically, particularly when presenting as a non-healing extraction socket with exposed necrotic bone. Persistent osteomyelitis-like lesions, especially in patients with established risk factors such as areca nut use, should prompt early biopsy to exclude underlying malignancy. This case underscores the importance of maintaining a high index of suspicion; correlating clinical, radiological, and histopathological findings; and adopting a multidisciplinary approach to facilitate timely diagnosis and appropriate oncologic management.

## Introduction

Osteomyelitis of the maxilla is an uncommon condition because of the rich vascularity and porous architecture of the maxillary bone, which generally confer resistance to infection. It most commonly develops secondary to odontogenic infections, trauma, systemic immunocompromised states, or following dental extractions. Clinically, patients may present with pain, swelling, purulent discharge, exposed necrotic bone, and non-healing extraction sockets [1-4]. Similar manifestations may also occur in advanced oral squamous cell carcinoma (SCC) [1,3-5], particularly when secondary infection and tumor-associated bone necrosis are present, creating a significant diagnostic challenge.

SCC is the most common malignancy of the oral cavity and is strongly associated with risk factors such as tobacco use, areca nut chewing, and alcohol consumption [6,7]. Although maxillary SCC is relatively uncommon compared with mandibular or tongue lesions, it frequently presents at an advanced stage because of its insidious onset and proximity to adjacent anatomical spaces, allowing early local extension into the maxillary sinus, nasal cavity, pterygopalatine region, and infratemporal fossa. Radiological findings may mimic chronic osteomyelitis [1,3,4] owing to extensive osteolytic destruction and inflammatory changes, potentially delaying diagnosis and definitive treatment.

We report a diagnostically challenging case of well-differentiated SCC of the maxilla presenting as a non-healing extraction socket with extensive osteomyelitis-like craniofacial bone destruction. The case emphasizes the importance of maintaining a high index of suspicion for underlying malignancy in persistent osteomyelitis-like lesions and highlights the indispensable role of early histopathological evaluation in establishing the diagnosis and guiding appropriate oncologic management.

## Case presentation

A 55-year-old woman presented to the Department of Oral and Maxillofacial Surgery with a three-month history of pain, swelling, and purulent discharge involving the left posterior maxillary region. The patient gave a history of extraction of the left maxillary molar tooth four months prior to presentation due to progressive tooth mobility. Following the extraction, the socket failed to heal and was associated with persistent pain, purulent discharge, progressive ulceration, and exposed necrotic bone. She also complained of mild restriction in mouth opening and difficulty in mastication.

The patient had a 20-year history of areca nut chewing, which she had discontinued one year before presentation. Her medical history was non-contributory, with no history of diabetes mellitus, hypertension, immunocompromised status, previous radiotherapy, or antiresorptive medication use.

**Figure 1:**
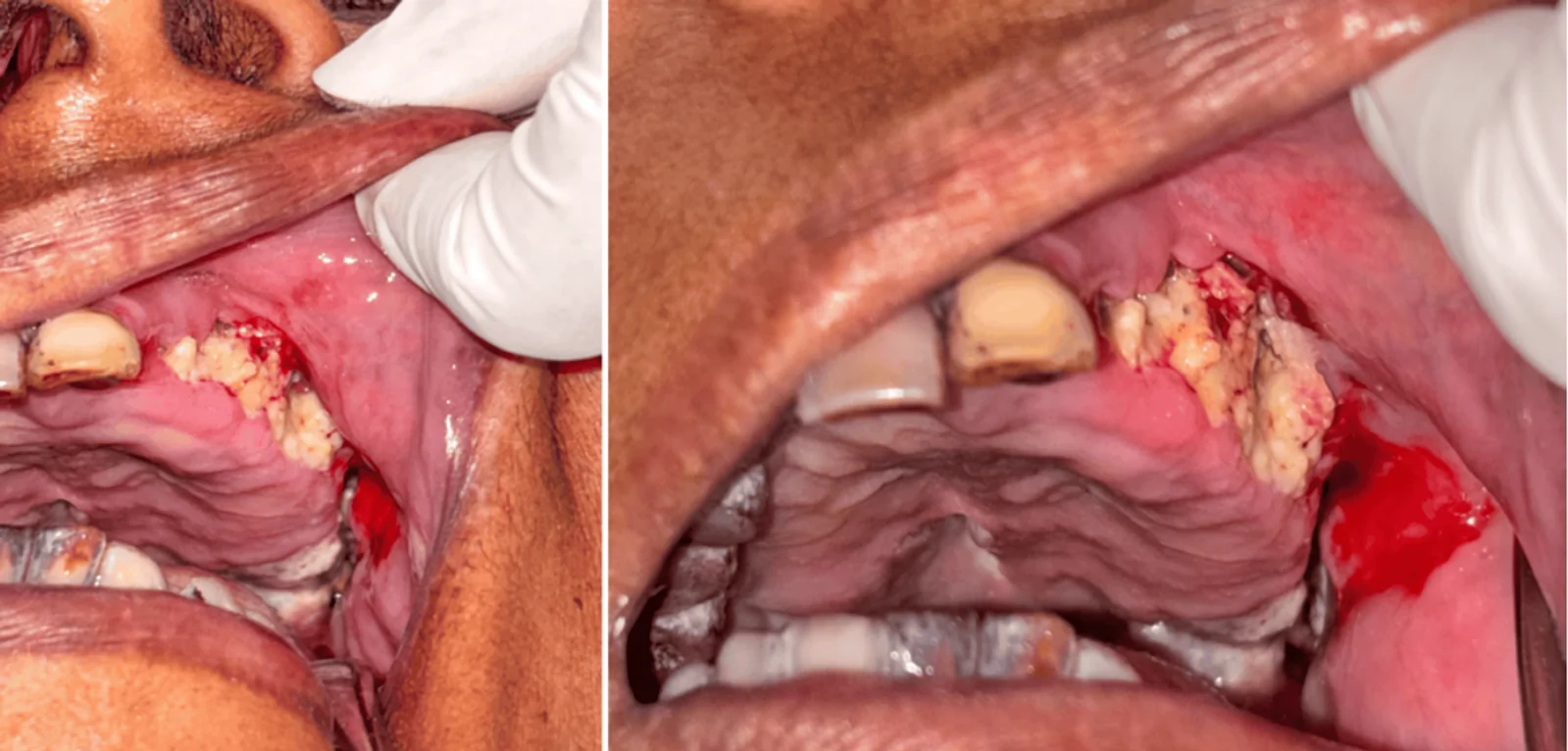
Intraoral photographs showing an ulcerative lesion with exposed necrotic tissue and irregular granulation tissue involving the left posterior maxillary alveolus and hard palate, associated with destruction of the adjacent alveolar ridge and maxillary tuberosity region.

Extraoral examination revealed a diffuse swelling over the left midfacial region involving the cheek, producing facial asymmetry. Mild restriction in mouth opening was noted. A single palpable left level IB lymph node was identified clinically; however, no radiologically significant cervical lymphadenopathy was demonstrated on contrast-enhanced computed tomography (CECT). Intraoral examination demonstrated an ulcerative lesion involving the left maxillary tuberosity and posterior maxillary alveolar region extending onto the hard palate (Figure 1).

The lesion exhibited irregular margins with exposed necrotic bone and friable necrotic tissue. A palatal perforation communicating with the underlying maxillary defect was also evident. Based on the history of a non-healing extraction socket, exposed necrotic bone, and purulent discharge, a provisional diagnosis of chronic osteomyelitis of the left maxilla was considered, with oral SCC included in the differential diagnosis. Routine hematological investigations were within normal limits.

**Figure 2:**
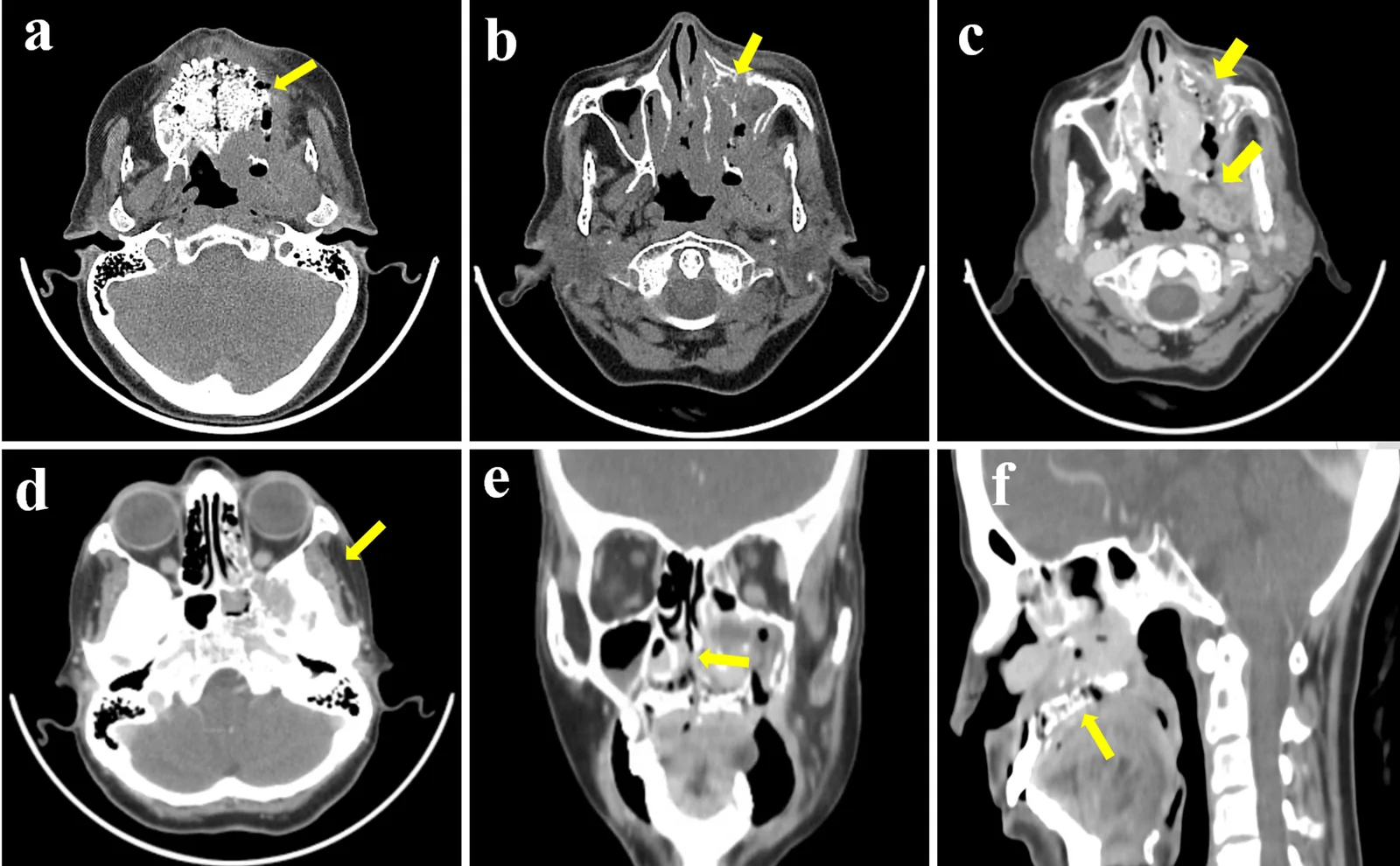
CECT of the head and neck. Axial CECT image demonstrating (a) an ill-defined enhancing soft-tissue lesion centered in the left maxilla with osteolytic destruction of the hard palate and maxillary alveolar process (yellow arrow), (b) complete opacification of the left maxillary sinus with destruction of the anterior, medial, lateral, and posterior sinus walls (yellow arrow), (c) extension of the lesion into the left nasal cavity with destruction of the lateral nasal wall and adjacent maxillary structures (yellow arrows), and (d) destruction of the left zygomaticomaxillary buttress with extension into the adjacent facial soft tissues (yellow arrow). (e) Coronal CECT image demonstrating erosion of the hard palate and left lateral nasal wall, with extension across the midline to involve the medial wall of the contralateral maxillary sinus (yellow arrow). (f) Sagittal CECT image demonstrating posterior extension of the lesion with destruction of the posterior wall of the maxillary sinus and involvement of the pterygopalatine region and infratemporal fossa (yellow arrow). CECT: contrast-enhanced computed tomography.

CECT of the head and neck revealed an ill-defined enhancing destructive lesion involving the hard palate, all walls of the left maxillary sinus, left pterygoid bone, left lateral wall of the nasal cavity, medial wall of the right maxillary sinus, and extension into the left infratemporal fossa and masticator space (Figure 2).

**Figure 3:**
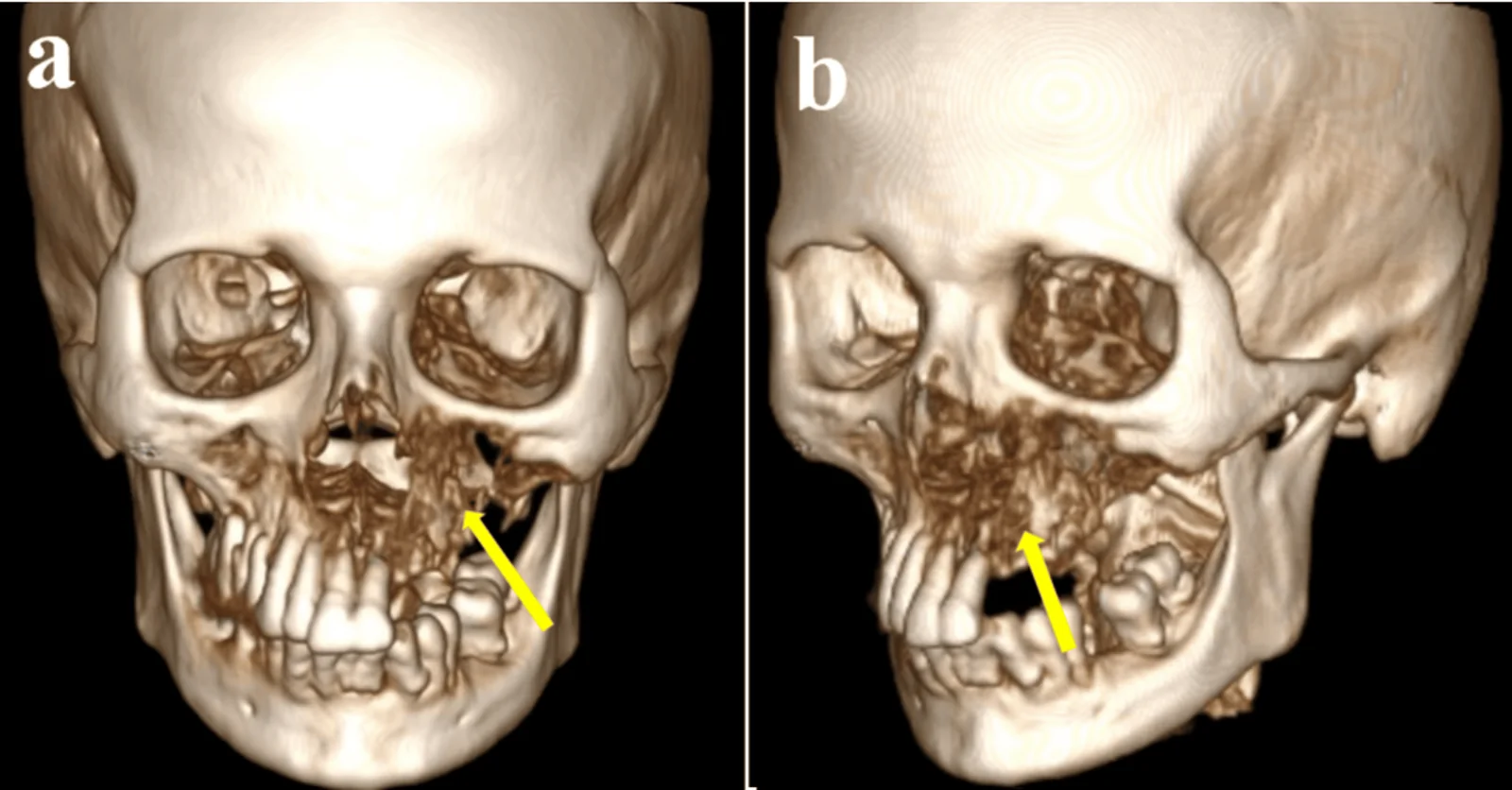
Three-dimensional reconstructed CT images of the facial skeleton. (a) Frontal view and (b) left anterolateral oblique view show extensive osteolytic destruction involving the left maxillary alveolar process, hard palate, anterior wall of the maxillary sinus, piriform aperture, and zygomaticomaxillary buttress (yellow arrow), resulting in marked loss of the left midfacial bony framework. CT: computed tomography.

Extensive osteolytic destruction of the left maxilla with associated soft tissue involvement was evident, and the radiological impression favored extensive osteomyelitis. Three-dimensional CT further demonstrated marked destruction of the left maxillary alveolus, hard palate, anterior maxillary wall, and zygomaticomaxillary buttress (Figure 3). No radiologically significant cervical lymphadenopathy was identified.

**Figure 4:**
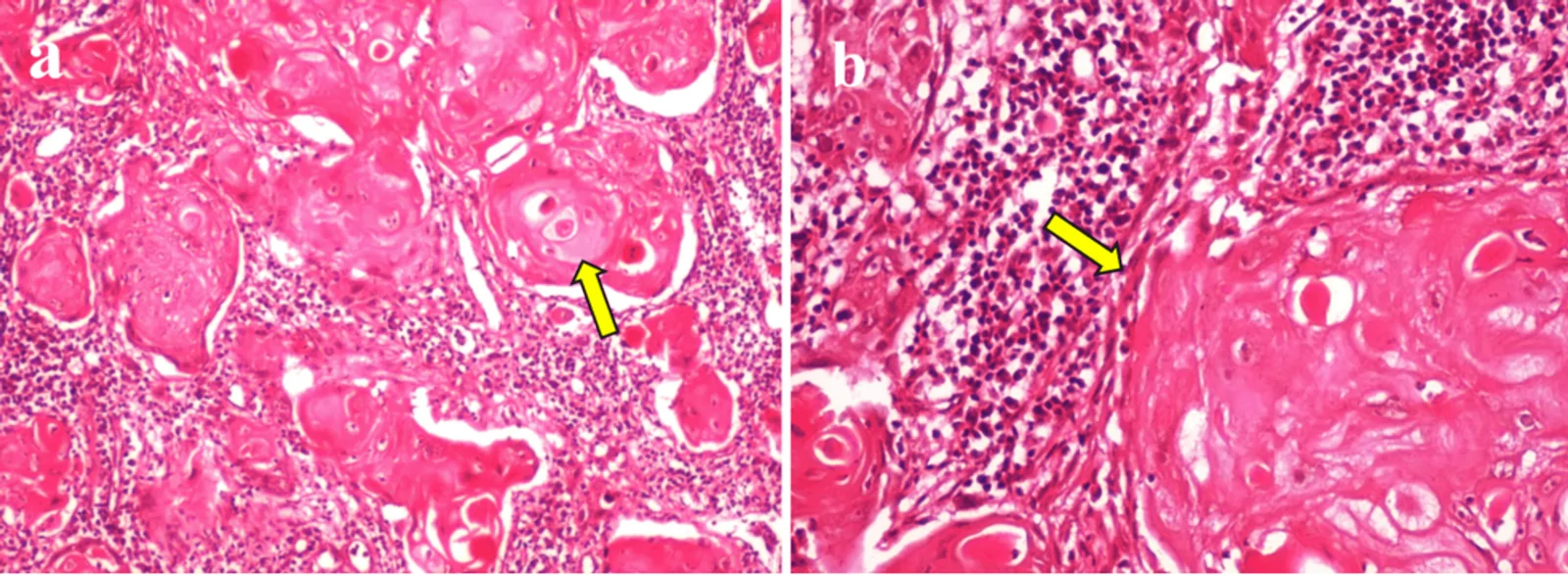
Histopathological examination (H&E stain). (a) Low-power view (×10) showing infiltrating nests of malignant squamous epithelial cells with keratin pearl formation (arrow). (b) High-power view (×40) demonstrating a keratin pearl (arrow) with surrounding malignant squamous epithelial cells exhibiting keratinization and nuclear pleomorphism, consistent with well-differentiated SCC. H&E: hematoxylin and eosin; SCC: squamous cell carcinoma.

An incisional biopsy was performed from the viable margin of the lesion. Histopathological examination revealed infiltrating nests and islands of malignant squamous epithelial cells invading the connective tissue stroma. The tumor cells exhibited abundant eosinophilic cytoplasm, hyperchromatic nuclei, mild-to-moderate nuclear pleomorphism, intercellular bridges, and focal keratin pearl formation, consistent with well-differentiated SCC (Figure 4).

Following multidisciplinary evaluation, the patient was referred to the Department of Medical Oncology. Based on the clinical and radiological findings, the lesion was staged as cT4bN0M0 (AJCC 8th edition) [8] owing to invasion of the pterygoid region and infratemporal fossa. Because of extensive involvement of the infratemporal fossa, pterygoid region, and adjacent skull-base structures, the lesion was considered initially unresectable; therefore, induction chemotherapy with the TPF regimen (docetaxel, cisplatin, and 5-fluorouracil) was initiated in view of the extensive locoregional disease. The patient remains under regular oncologic follow-up for reassessment and planning of further definitive management. At the latest follow-up (three months after induction chemotherapy), the patient demonstrated symptomatic improvement with reduction in pain and mild regression of the lesion. The chronological sequence of clinical events from presentation to diagnosis and management is summarized in Table 1.

**Table 1:** Timeline of clinical events from presentation to diagnosis and management. CECT: contrast-enhanced computed tomography; SCC: squamous cell carcinoma.

Timeline	Clinical event
Three months before presentation	Patient developed pain and mobility of the left maxillary second molar associated with a gradually progressive swelling over the left midface.
Four months before presentation	Extraction of the mobile left maxillary second molar was performed at a local dental clinic. The extraction socket failed to heal, with persistent pain, purulent discharge, and progressive facial swelling.
Presentation to our institution	Clinical examination revealed diffuse left midfacial swelling, facial asymmetry, intraoral necrotic exposed bone involving the left posterior maxilla, a non-healing extraction socket, and purulent discharge, suggestive of chronic osteomyelitis.
Initial investigations	CECT demonstrated an ill-defined enhancing soft-tissue lesion centered in the left maxilla with extensive osteolytic destruction involving the hard palate, maxillary sinus walls, zygomaticomaxillary buttress, lateral nasal wall, pterygoid region, and extension into the infratemporal fossa.
Definitive diagnosis	Incisional biopsy was performed. Histopathological examination confirmed well-differentiated SCC.
Staging	The patient was staged as cT4bN0M0 (AJCC 8th edition). The tumor was considered initially unresectable because of posterior extension into the infratemporal fossa and pterygoid region.
Multidisciplinary management	Following multidisciplinary tumor board discussion, induction chemotherapy with the TPF regimen (docetaxel, cisplatin, and 5-fluorouracil) was initiated.
Follow-up	After three cycles of chemotherapy, the patient demonstrated partial clinical improvement, with reduction in pain and facial swelling and mild regression of the lesion. She remains under regular oncologic surveillance for reassessment and planning of definitive treatment.

## Discussion

SCC accounts for approximately 90% of all oral malignancies and remains a significant cause of morbidity and mortality worldwide [7]. Although the tongue, floor of the mouth, and buccal mucosa are the most frequently affected sites, SCC involving the maxilla is relatively uncommon and often presents at an advanced stage because of the anatomical complexity of the maxillary region and the nonspecific nature of early clinical manifestations. The close proximity of the maxilla to the maxillary sinus, nasal cavity, pterygopalatine fossa, infratemporal fossa, and skull base permits early local extension before obvious clinical symptoms develop.

This case illustrates an unusual presentation of advanced maxillary SCC masquerading as chronic osteomyelitis. The patient initially presented with pain, swelling, purulent discharge, a non-healing extraction socket, exposed necrotic bone, and palatal perforation following extraction of a mobile maxillary molar. These findings, together with extensive osteolytic destruction on CECT, strongly suggested chronic osteomyelitis. Osteomyelitis of the maxilla is uncommon due to its abundant vascularity and collateral blood supply; however, when present, it is usually associated with odontogenic infections [6], trauma, uncontrolled diabetes, immunocompromised states, or previous irradiation. None of these predisposing systemic factors were identified in our patient, making the extensive osteomyelitis-like appearance particularly unusual.

A non-healing extraction socket is a well-recognized but frequently overlooked manifestation of oral SCC [1,3,4]. Malignant infiltration of the alveolar bone may result in progressive tooth mobility, leading to extraction under the assumption of periodontal or odontogenic disease [4,5]. Failure of the extraction socket to heal, persistent pain, exposed bone, progressive ulceration, or recurrent discharge should prompt immediate histopathological evaluation [1,5] to exclude an underlying malignancy. In this case, the extracted tooth had become progressively mobile because of the underlying disease process, and the subsequent non-healing socket further contributed to the initial clinical impression of osteomyelitis.

Radiologically, distinguishing chronic osteomyelitis from malignant bone destruction can be challenging. Chronic osteomyelitis typically demonstrates irregular osteolysis, cortical destruction, sequestrum formation, periosteal reaction, and surrounding inflammatory soft tissue changes. In contrast, SCC usually produces an ill-defined invasive osteolytic lesion with cortical erosion and an associated enhancing soft tissue mass. Nevertheless, advanced SCC complicated by secondary infection and extensive bone necrosis may closely resemble osteomyelitis on cross-sectional imaging, as observed in this case. The CECT demonstrated destruction of the hard palate, all walls of the left maxillary sinus, the left pterygoid bone, the left lateral wall of the nasal cavity, the medial wall of the contralateral maxillary sinus, and extension into the infratemporal fossa. Despite this extensive involvement, the radiological impression favored osteomyelitis, highlighting the limitations of imaging alone in establishing the diagnosis.

Histopathological examination remains the gold standard for differentiating malignant lesions from chronic inflammatory conditions [2,7]. Incisional biopsy in our patient demonstrated infiltrating nests and islands of malignant squamous epithelial cells with keratinization and intercellular bridges, confirming well-differentiated SCC. This finding completely altered the therapeutic approach from management of presumed osteomyelitis to multidisciplinary oncologic care.

The patient had a long-standing history of areca nut chewing for approximately 20 years, a well-established risk factor for oral SCC. Areca nut induces chronic mucosal injury, epithelial dysplasia, oxidative stress, and genomic instability, thereby increasing the risk of malignant transformation [6,7]. Although the patient had discontinued the habit one year before presentation, the cumulative carcinogenic exposure likely contributed to the disease development. This underscores the importance of carefully evaluating persistent oral lesions in individuals with known carcinogenic habits, irrespective of whether the habit has been discontinued.

Management of advanced maxillary SCC requires a multidisciplinary approach involving oral and maxillofacial surgeons, head and neck surgeons, medical oncologists, radiation oncologists, radiologists, and pathologists. Following discussion at the multidisciplinary tumor board, the lesion was considered initially unresectable because of invasion of the infratemporal fossa, pterygoid region, and adjacent skull-base structures. Therefore, induction chemotherapy using the TPF regimen (docetaxel, cisplatin, and 5-fluorouracil) was initiated. Following three cycles of chemotherapy, the patient demonstrated partial clinical improvement, with reduction in symptoms and mild regression of the lesion, and continues under oncologic surveillance for further treatment planning.

Only a limited number of published reports have described oral SCC presenting clinically as chronic osteomyelitis or a non-healing extraction socket [1,3-5]. Most reported cases involve the mandible, whereas maxillary involvement with such extensive craniofacial extension is considerably less common. Compared with previously reported cases, our patient demonstrated far more extensive craniofacial destruction involving the hard palate, maxillary sinus, pterygoid bone, contralateral maxillary sinus, and infratemporal fossa. Unlike many previously reported cases in which imaging suggested malignancy, the radiological appearance in our patient strongly favored osteomyelitis, delaying the diagnosis until histopathological confirmation. This unusual clinicoradiological discordance makes the present case particularly noteworthy. Persistent osteomyelitis-like lesions of the maxilla, particularly those associated with tooth mobility, non-healing extraction sockets, or established carcinogenic habits, should undergo early biopsy to exclude an underlying malignancy.

A limitation of this report is the relatively short follow-up after induction chemotherapy, precluding assessment of long-term oncologic outcome. Nevertheless, this case highlights an important diagnostic pitfall in the evaluation of persistent osteomyelitis-like lesions of the maxilla and the need for clinicians to maintain a high index of suspicion when managing these cases, particularly in patients with established risk factors for oral cancer. Previously reported cases of SCC presenting as osteomyelitis or a non-healing extraction socket are summarized in Table 2.

**Table 2:** Previously reported cases of SCC presenting as osteomyelitis or a non-healing extraction socket and the present case. MRONJ: medication-related osteonecrosis of jaw; TPF: docetaxel (Taxotere), cisplatin (Platinol), and fluorouracil (5-FU); SCC: squamous cell carcinoma.

Author (year)	Patient demographics	Site	Initial clinical diagnosis	Radiological findings	Final diagnosis	Treatment	Clinical significance
Vezeau et al. (1990) [4]	Adult-male	Mandible	Chronic osteomyelitis	Osteolytic mandibular destruction with cortical erosion	Invasive SCC	Surgical resection	One of the earliest reports demonstrating SCC presenting clinically as chronic osteomyelitis of the mandible
Mauceri et al. (2021) [1]	Three elderly patients	Maxilla and mandible	MRONJ	Exposed bone with nonspecific osteolytic changes and bone destruction	Oral SCC	Biopsy followed by definitive oncologic management	Case series highlighting oral SCC masquerading as MRONJ and emphasizing the importance of histopathological examination
Ghosh et al. (2025) [3]	58-Year-old-male	Mandible	Chronic osteomyelitis	Extensive mandibular osteolysis with associated soft-tissue involvement	Well-differentiated SCC	Surgical resection with oncologic management	SCC closely mimicked chronic osteomyelitis both clinically and radiographically, resulting in delayed diagnosis
Unni et al. (2025) [5]	Middle-aged adult	Mandible	Dentoalveolar abscess/non-healing extraction socket	Periapical osteolytic lesion with cortical destruction	Oral SCC	Surgical management	SCC initially presented as a dentoalveolar abscess, highlighting the importance of biopsy in persistent extraction-site lesions
Present case	55-Year-old-female	Left posterior maxilla	Extensive chronic osteomyelitis following extraction of a mobile maxillary molar	Extensive osteolytic destruction involving the hard palate, all walls of the left maxillary sinus, left pterygoid bone, lateral nasal wall, and the medial wall of the contralateral maxillary sinus, with extension into the infratemporal fossa	Well-differentiated SCC	Induction chemotherapy with TPF for initially unresectable disease	Extensive maxillary SCC with infratemporal fossa extension radiologically mimicking chronic osteomyelitis, resulting in delayed diagnosis until histopathological confirmation

## Conclusions

This case highlights the diagnostic challenge posed by advanced maxillary SCC masquerading as chronic osteomyelitis following tooth extraction. The combination of a non-healing extraction socket, exposed necrotic bone, palatal perforation, and extensive craniofacial bone destruction initially favored an infective process; however, histopathological examination established the diagnosis of well-differentiated SCC. Clinicians should maintain a high index of suspicion for malignancy in persistent osteomyelitis-like lesions, particularly in patients with oral cancer risk factors. Early biopsy remains the cornerstone of diagnosis and facilitates timely multidisciplinary intervention, thereby minimizing delays in definitive treatment.
